# Antineoplastic and Immunomodulatory Effects of Cabozantinib in Continuous and Primary Human Anaplastic Thyroid Cancer Cells †

**DOI:** 10.3390/cancers18152379

**Published:** 2026-07-23

**Authors:** Giusy Elia, Silvia Martina Ferrari, Eugenia Balestri, Chiara Botrini, Francesca Ragusa, Valeria Mazzi, Licia Rugani, Simona Piaggi, Elena Catania Romizi, Oriana Fabrazzo, Eleonora Giorgetti, Alessia Baglini, Gilda Varricchi, Camilla Virili, Salvatore Ulisse, Gabriele Materazzi, Alessandro Antonelli, Poupak Fallahi

**Affiliations:** 1 Department of Surgical, Medical and Molecular Pathology and Critical Area, University of Pisa, 56126 Pisa, Italy; giusy.elia@unipi.it (G.E.); silvia.ferrari@unipi.it (S.M.F.); eugenia.balestri@phd.unipi.it (E.B.); chiara.botrini@med.unipi.it (C.B.); francesca.ragusa@phd.unipi.it (F.R.); valeria.mazzi@ao-pisa.toscana.it (V.M.); licia.rugani99@gmail.com (L.R.); elena.catr@gmail.com (E.C.R.); oriana.fabrazzo91@gmail.com (O.F.); alessia.baglini@gmail.com (A.B.); gabriele.materazzi@unipi.it (G.M.); 2 Department of Translational Research and New Technologies in Medicine and Surgery, University of Pisa, 56126 Pisa, Italy; simona.piaggi@unipi.it (S.P.); eleonora.giorgetti.me@gmail.com (E.G.); poupak.fallahi@unipi.it (P.F.); 3 Department of Translational Medical Sciences and Center for Basic and Clinical Immunology Research (CISI), University of Naples Federico II, 80131 Naples, Italy; gilda.varricchi@unina.it; 4 World Allergy Organization (WAO) Center of Excellence, 80131 Naples, Italy; 5 Institute of Experimental Endocrinology and Oncology (IEOS), National Research Council, 80131 Naples, Italy; 6 Department of Medico-Surgical Sciences and Biotechnologies, Endocrinology Section, “Sapienza” University of Rome, 04100 Latina, Italy; camilla.virili@uniroma1.it; 7 Department of Surgery, “Sapienza” University of Rome, 00161 Rome, Italy; salvatore.ulisse@uniroma1.it

**Keywords:** anaplastic thyroid cancer, cabozantinib, primary cell cultures, in vitro study

## Abstract

**Background/Objectives:** To date, the antineoplastic effect of cabozantinib in in vitro anaplastic thyroid cancer (ATC) cells has not yet been investigated. We aim to evaluate the antineoplastic and immunomodulatory effect of cabozantinib in both primary and continuous ATC cell cultures in vitro. **Methods:** The antineoplastic activity of cabozantinib was evaluated in ATC cell lines (8305C; CAL62) and in five different primary ATC cells obtained directly from patients. **Results:** Increasing concentrations of cabozantinib were tested showing a significant decrease in viability/proliferation and an increase in apoptosis in the treated cells vs. control. A scratch assay was also performed to evaluate the effect of cabozantinib on migration showing a significant inhibition of it vs. control. Moreover, we showed a decrease in the secretion of both CXCL10 and CCL2 (T-helper (Th)1 and (Th)2 chemokines) under the treatment with cabozantinib, thus suggesting an immunomodulatory effect of the drug. **Conclusions:** A personalized approach for each patient can be achieved by using primary human ATC cells obtained directly from the patient, since in vitro studies permit assessing the chemosensitivity of cells obtained from each subject. These results in our opinion are very promising for a future in vivo evaluation of cabozantinib in patients with ATC.

## Simple Summary

Anaplastic thyroid cancer (ATC) is a very aggressive tumor, whose treatment is still a challenge. We aimed to investigate the tyrosine kinase inhibitor cabozantinib (that has been approved for the treatment of locally advanced or metastatic differentiated thyroid cancer) in cell cultures of ATC in an in vitro study. No study in the literature has evaluated the antineoplastic and immunomodulatory effects of cabozantinib in vitro in primary and/or continuous ATC cells. Therefore, we have performed in vitro studies in primary human cells of ATC (pATC) obtained directly from five patients and in two continuous ATC cell lines. It is noteworthy that pATC are able to maintain the features of the original tissues from which they derived therefore allowing a personalization of the therapy. Our results showed a significant antineoplastic activity and an immunomodulatory effect of cabozantinib in both continuous and primary human ATC cells.

## 1. Introduction

Thyroid carcinoma (TC) incidence is increasing worldwide, mainly due to the rising prevalence of papillary thyroid carcinoma, the most common histological subtype, which is generally associated with a favorable prognosis [1,2]. In contrast, anaplastic TC (ATC) is a rare disease, with an estimated incidence of fewer than six cases per 100,000 population in Europe and approximately 0.12–0.2 cases per 100,000 population in the United States [2]. ATC originates from the follicular epithelium and accounts for approximately 1.7% of all TC cases; it is a very aggressive malignancy characterized by a median survival of 6 months or less after diagnosis and a markedly poor quality of life [3,4]. ATC is characterized by: (a) a rapid growth, early metastases, which are present in half of cases at the diagnosis, mainly in pulmonary sites, as well as in the brain [4,5,6], and (b) resistance to conventional therapies. Standard management includes surgical debulking, accelerated hyper-fractionated “external beam radiation therapy”, and chemotherapy, but also combining these different therapeutics approaches; most of the studies showed a limited or null benefit on the survival of patients [4].

Emerging therapeutic strategies include novel targeted therapy and immunotherapy [4,7], with growing use of immune checkpoint inhibitors (ICIs), either as monotherapy or in combination with tyrosine kinase inhibitors (TKIs) [8,9]. Tumor treatment is becoming increasingly personalized through molecular and genetic profiling, which helps guide the selection of the most effective therapeutic approach. The molecular mutations found in ATC patients involved the *BRAF* [2,4] and *MEK* genes [10]; other mutations are related to *TP53*, *RET*, *RAS*, *TERT* promoter, *PIK3CA*, *EIF1AX*, *PTEN*, *NES*, *IQGAP1*, *CCND1*, *MCL1*, *POU5F1*, *MYBL2*, *SOX2*, and *NANOG* [2]. Targeted therapies have become a good alternative for subjects harboring *BRAF*, *RET*, *NTRK*, *ALK*, and *ROS1* alterations with efficacy reported even in ATC [11]. The multikinase TKI (mTKI) sorafenib, lenvatinib, and cabozantinib are approved for patients with metastatic radioiodine-refractory differentiated TC (RAIR-DTC) [11,12,13]. The mTKI sorafenib was the first approved for RAIR-DTC, followed by the approval of lenvatinib and cabozantinib [14]. Sorafenib showed significant results in inhibiting ATC cells growth in vitro and in improving the survival rate of xenograft mouse model of ATC [14,15]. However, a phase II trial in advanced ATC patients showed a limited efficacy of the drug reporting a median progression free survival (PFS) of 1.9 months, a one-year survival rate of 20%, and a partial response (PR) in 2/20 patients [16]. In a clinical trial, ATC patients in treatment with sorafenib reported a median PFS of 2.8 months and a median overall survival (OS) of 5 months [17]. Lenvatinib has shown its ability in inhibiting the proliferation of ATC cells both in in vitro and in vivo studies [18]. However, lenvatinib as single agent does not show a significant efficacy in ATC. In a multicenter single-arm phase 2 trial, 3% of patients had a PR and 50% achieved stable disease (SD) (≥5 weeks). Despite this, patients reported a short duration of the response with a median PFS of 2.6 months and an OS of 3.2 months [19]. Actually, lenvatinib is approved in Japan in patients with unresectable ATC [20]. Recently, a study [21] investigated the efficacy of lenvatinib in patients with unresectable ATC and showed an OS rate of 11.9% at 1 year, an objective remission rate of 11.9%, and a clinical benefit rate of 33.3%; the number of responders was low but the response was sustained. However, it has been observed that the antineoplastic effects of lenvatinib can be improved through combination with anti-PD1/PD-L1 agents, vinorelbine, or paclitaxel [22,23]. Other types of combinations involved dabrafenib and trametinib, which showed an important and significant clinical effect in patients with ATC carrying a *BRAF^V600E^* mutation, with an overall response rate (ORR) of 69%, a prolonged duration of response, and survival with controllable toxicity [24]. Therefore, the FDA approved the combination of these drugs for patients with *BRAF^V600E^*-mutant ATC that has metastasized or is inoperable. Recently, the phase II open-label study [25] confirmed the substantial survival benefit of the combination of dabrafenib and trametinib in patients with ATC. At the time of the data cut-off, after approximately six years of follow-up, the study reported an ORR of 56%, including three complete responses, while the 12-month duration of response rate was 50%. The BRAF and MEK inhibitor combination has significantly improved the outcomes of ATC patients with *BRAF^V600E/K^* mutations. A promising combination is also represented by lenvatinib with pembrolizumab, which shows significant synergy in ATC. Encouraging results have been showed in a phase 2 trial on ATC and poorly DTC (PDTC) patients [26]. Indeed, ATC patients showed a 66% of complete remissions, 16% SD, and 16% progressive disease (PD), and the median PFS was of 16.5 months for ATCs [26]. Cabozantinib is a promising drug acting against several key receptors implicated in TC growth, angiogenesis, and metastatic progression. Its targets are MET and VEGF receptors, AXL, RET, MER, ROS1, TYRO3, TRKB, KIT, FLT3, and TIE-2 [27]. Lately, cabozantinib was approved by FDA and European Medicines Agency as treatment for adult patients with locally advanced or metastatic DTC, not eligible or refractory to RAI (progressed during or after prior systemic therapy) [27,28]. The promising outcome of cabozantinib has been shown in the multicenter trial COSMIC-311 involving patients with locally advanced or metastatic DTC (RAI-refractory or not eligible) that had progressed after up to two prior vascular endothelial growth factor (VEGFR)-targeting therapies. Indeed, in the cabozantinib group, a significant reduction in disease progression or death was observed with respect to the placebo group and the median PFS was significantly longer in the cabozantinib arm. These results suggested the use of cabozantinib as new therapy for patients with RAIR-DTC without a standard of care, with a daily recommended dosage of 60 mg [29,30]. Moreover, the authors performed a subgroup analysis of COSMIC-311, aiming at investigating the role of *BRAF^V600E^* mutation in the outcome of response to treatment with cabozantinib, showing an improved ORR and PFS with respect to placebo regardless of the *BRAF* mutation status [31]. Recently, a retrospective study on seven patients with PDTC who received cabozantinib (most of them were previously treated with other first-line TKIs) showed that this could be a valid option for those patients who have progressed or cannot tolerate other TKIs (lenvatinib, sorafenib). Actually, cabozantinib showed a high response to the therapy with a PR plus SD of 86% and a median PFS of 12.9 months, and patients also had good tolerability to the drug [32]. Ongoing research efforts are aimed at elucidating the optimal use of cabozantinib in different subtypes of TC and identifying biomarkers predictive of treatment response [33]. Recently some studies have showed that human ATC cells are able to secrete cytokines and chemokines that are involved in the immune response against the tumor [34,35]. Furthermore, immunotherapy has been demonstrated to be effective in patients with ATC, also showing an immunomodulatory effect on cytokine and chemokine secretion [36,37,38].

To date, the antineoplastic effect of cabozantinib in in vitro ATC cell studies has not yet been investigated; furthermore, no study has evaluated if cabozantinib is able to immunomodulate the Th1 or Th2 chemokine secretion by ATC cells, which are involved in the immune response to the tumoral cells. Therefore, we aim to investigate the antineoplastic and immunomodulatory effects of cabozantinib both in human primary and continuous cell cultures of ATC in vitro.

## 2. Materials and Methods

### 2.1. Reagents and Drugs

Cabozantinib (Aurogene) was dissolved in 100% dimethyl sulfoxide (DMSO) to obtain a 10 mmol/L stock solution for in vitro experiments. DMSO, at the highest concentration, used for drug dilution, served as the vehicle control.

DMEM, RPMI, and all other reagents were purchased from Sigma-Aldrich (Merck, Darmstadt, Germany), whereas reagents for quantitative real-time PCR were obtained from Applied Biosystems–Life Technologies (Milan, Italy) [39].

### 2.2. Anaplastic Thyroid Cancer Cells

The continuous ATC cell lines 8305C and CAL62 were purchased from DSMZ German Collection.

8305C cells were cultured in RPMI-1640 supplemented with 10–20% FBS and 2 mM L-glutamine, whereas CAL62 cells were cultured in DMEM supplemented with 5–10% FBS and 2 mM L-glutamine. Both cell lines were maintained at 37 °C in a humidified incubator with 5% CO_2_ [40].

### 2.3. Human Primary Anaplastic Thyroid Cancer Samples

The surgical thyroidal samples of three females and two males with ATC were used to obtain human primary cells (Table 1). At the time of the first surgical operation patients had a tumor size range of 6–14 cm, and the diagnosis is in line with the accepted laboratory, histological, and clinical criteria [39,41]. Four out five patients were classified as T4 as per thyroid malignancy World Health Organization (WHO) classification and the sixth edition of tumor–node–metastasis (TNM) system, and according to the more recently TNM guidelines 8th any T is accepted for ATC [42]. We identified known mutations (*BRAF*, RET/PTC1, RET/PTC3, *N-RAS*, *K-RAS*, *H-RAS*, and *PAX8/PPARg*) through PCR single-strand conformation polymorphism and direct DNA sequencing and we confirmed through immunohistochemistry (IHC) the absence of thyroperoxidase (TPO), thyroglobulin (Tg), thyroid-stimulating hormone (TSH) receptor, and sodium/iodide symporter (NIS) expression [39]. We obtained the consensus to take part in the study from all patients with the approval from the Ethics Committee of the University of Pisa.

### 2.4. Primary Anaplastic Thyroid Cancer Cell Culture

Five different primary ATC cells were obtained from the tumoral fragments that were finely minced, washed in M-199 medium supplemented with 500,000 U/L penicillin, 500,000 U/L streptomycin, and 1,000,000 U/L nystatin, and then maintained in a DMEM medium with 10% fetal calf serum (37 °C, 5% CO_2_). At the third passage, cells were seeded in Methocel to assess colony-forming efficiency, and the largest colonies were subsequently expanded in culture flasks [39]. Once cells reached the fourth passage we performed the chemosensitivity tests in line with the previously published studies [43]. The absence of TPO, Tg, TSH receptor and NIS expression was assessed through IHC, which showed a partial and focal positivity of cytokeratin instead. DNA fingerprinting showed a pattern identical to the original neoplastic tissue [39].

Two primary ATC cells harbor the *BRAF^V600E^* mutation, one primary ATC cell harbors the *NRAS* mutation, whereas no RET/PTC1 and RET/PTC3 rearrangements were detected through real-time PCR.

### 2.5. Cell Proliferation Assays

Cell proliferation was assessed using the WST-1 assay (Roche Diagnostics, Monza, Italy) [44]. Continuous ATC cell lines and primary human ATC cells were seeded into 96-well microtiter plates at a density of 35,000 cells per well in 100 μL of culture medium.

The cells were incubated with cabozantinib (1, 5, 10, 50 and 100 μM) or with vehicle alone for 2 days in an incubator under a humidified atmosphere (37 °C, 5% CO_2_). Cell proliferation was evaluated by measuring absorbance at 450 nm using a spectrophotometer. Untreated cells served as controls, while wells containing culture medium and WST-1 reagent without cells were used as background controls. Controls were normalized to 100% for each assay, and treatments were expressed as a percentage of the control. We measured the absorbance two times starting from tetrazolium reaction, after 1 and 2 h. The experiments were performed three times in each cell line and also in each human primary cell culture, and the mean percentage of inhibition of proliferation was calculated with respect to the control for each sample.

The proliferation was also assessed using the cell number counting, as previously published [39].

### 2.6. Apoptosis

#### 2.6.1. The Hoechst Assay

To perform the apoptosis assay we seeded primary ATC cells and continuous cell lines into 96-well microtiter plates at a density of 35,000 cells per 100 μL of culture medium. The incubation with cabozantinib (1, 5, 10, 50 and 100 μM) at 37 °C, 5% CO_2_ lasted 2 days. At the end of the treatment the cells were stained with 5 mg/mL of Hoechst 33342 (Sigma-Aldrich) for 10 min at 37 °C, as previously reported [39]. The apoptotic cells incorporating the Hoechst dye/total cells ×100 ratio (apoptosis index) were evaluated [39].

#### 2.6.2. Annexin V Binding Test

The Annexin V staining was also performed, as previously described [39]. Human primary ATC cells and continuous cell lines treated with cabozantinib were seeded for 2 days in a Lab-TekII Chamber Slide System (Nalge Nunc International, ThermoFisher Scientific, Waltham, MA, USA), and then they were observed under a fluorescence microscope [39].

#### 2.6.3. Scratch Test

We assessed cell migration by performing a scratch test. Primary ATC cells, and continuous cell lines were seeded in a 24-well microtiter plate at a concentration of 150,000 cells/500 μL. Once confluence was reached, the monolayer was firstly washed with PBS to remove detached cells, and then a serum-free medium was added.

After 1 h of incubation the scratch was performed by using a 200 μL sterile micropipette and subsequently the culture medium, in presence of 5% FBS with or without cabozantinib (1 μM), was added. The experiment was conducted with the lower concentration of cabozantinib, which was sufficient to inhibit the migration of the cells.

Plates were observed after 24 h under an inverted optical microscope equipped with a digital camera. Cells were photographed, and cell migration was assessed through gap width measurement using software ImageJ bundled with Java 8.

#### 2.6.4. Western Blotting

Immunoblot analysis was performed as previously described [43]. Human primary cells were treated with 50 μM cabozantinib for 1, 2 and 4 h, and then they were washed with PBS and collected with lysis buffer. Immunodetection was done employing antibodies against p-extracellular signal-regulated kinase (ERK)1/2, p-Akt (ser473), p-Akt (thr308), Akt, and ERK-1/2 (Cell Signaling Technology, Danvers, MA, USA). Detection was obtained using peroxidase-labeled anti-rabbit IgG and BM Chemiluminiscence Blotting Substrate (peroxidase) (Roche Diagnostics S.p.A, Milan, Italy). The acquisition and analysis of images were performed with a Chemi-Doc apparatus (Bio-Rad, Segrate, Italy).

Moreover, we have performed a Western blotting analysis, evaluating the effect of cabozantinib on Bax, confirming data obtained with the Hoechst and annexin V binding assays.

### 2.7. ELISA for CXCL10 and CCL2

We also aimed to evaluate a possible immunomodulatory effect of cabozantinib on the chemokine secretion in ATC cells focusing in particular on the T-helper (Th)1 and (Th)2 prototype chemokines, namely CXCL10 and CCL2. Indeed, chemokines are highly involved in the tumorigenesis as they are able to amplify the inflammatory and tumorigenic process, and both Th1 and Th2 chemokines are found to be increased in ATC [34,35,45].

The levels of the chemokines CXCL10 and CCL2 were dosed in the supernatants obtained from the cell cultures treated with cabozantinib (1, 5, 10, 50 and 100 μM), by using commercially prepared kits (R&D Systems, by Merk Life Science S.r.l., Milan, Italy). As control we used the supernatants from the cell cultures that were not treated. All samples were assessed in triplicate. The minimum (mean) detectable doses were 1.7 pg/mL for CCL2 and 1.67 pg/mL for CXCL10. The intra- and inter-assay coefficients of variation were 4.5 and 5.9%, respectively, for CCL2, while for CXCL10 they were 3.1 and 6.7%, respectively. The absorbance was measured at 450 nm using a spectrophotometer.

### 2.8. Statistical Analysis

The experiments with primary and continuous cells were conducted three times, in each cell line and also in each human primary cell culture. Therefore, for each human primary cell culture (from different donors) we report the results of the mean of three different experiments; we also report the mean of the results of the experiments conducted three times for each continuous cell line. The values are expressed as mean ± SD for normally distributed variables, otherwise as median and interquartile range. Differences among groups were analyzed using one-way ANOVA for normally distributed data. Proportions were compared using the χ^2^ test. Post hoc analyses were performed using the Bonferroni–Dunn and Tukey tests. Apoptosis data were analyzed through one-way ANOVA, followed by the Newman–Keuls multiple-comparison test. Statistical analysis were performed using StatView (version 5.0) and GraphPad Prism (version 10.5). A *p* value < 0.05 was considered statistically significant.

## 3. Results

### 3.1. Cabozantinib Significantly Inhibited Continuous and Human Primary ATC Cell Proliferation

**Figure 1:**
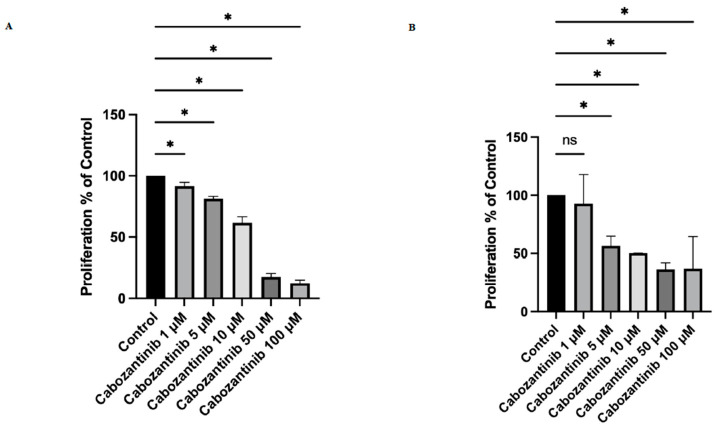
(**A**) Proliferation assay in CAL62 cells. The IC50 was 18.64 µM (through nonlinear regression). (**B**) Proliferation assay in 8305C cells. Bars represent the mean ± SD. ns: not significant (*p* > 0.05) * *p* < 0.05 vs. control as per Bonferroni–Dunn test. The IC50 was 3.30 µM (through nonlinear regression).

Both 8305C and CAL62 were treated with increasing concentrations of cabozantinib (1, 5, 10, 50 and 100 μM) for two days. A dose-dependent antiproliferative effect of cabozantinib was shown in CAL62 cells, with respect to control (*p* < 0.05) (Figure 1A), as well as in 8305C cells, with respect to control (*p* < 0.05) (Figure 1B). In the graphs we have reported the mean of the results of the experiments performed three times in 8305C as well as in CAL62.

**Figure 2:**
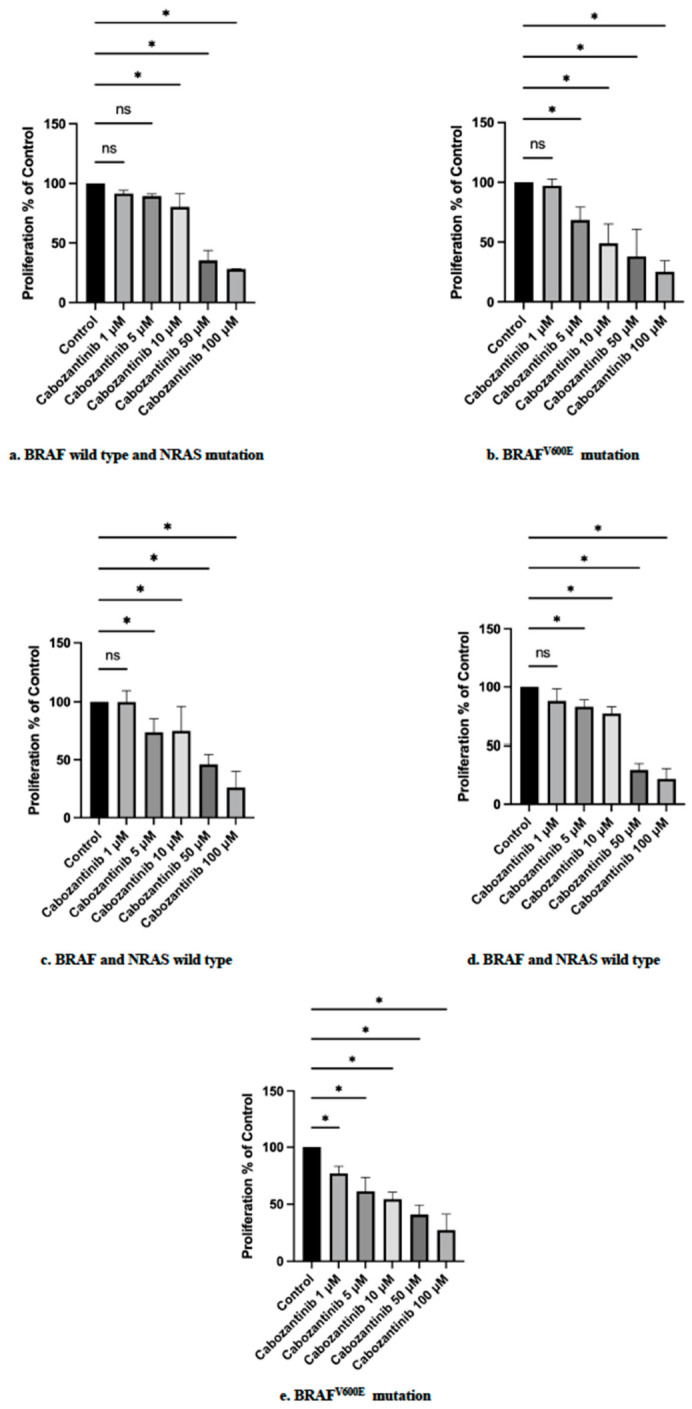
(**a**) Proliferation assay in the first human primary ATC cell culture with *BRAF* wild-type and *NRAS* mutation. The IC50 was 36.04 µM (through nonlinear regression). (**b**) Proliferation assay in the second human primary ATC cell cultures with *BRAF^V600E^* mutation. The IC50 was 6.16 µM (through nonlinear regression). (**c**) Proliferation assay in the third human primary ATC cell cultures with no reported mutations among the ones evaluated. The IC50 was 19.90 µM (through nonlinear regression). (**d**) Proliferation assay in the fourth human primary ATC cell cultures with no reported mutations among the ones evaluated. The IC50 was 32.22 µM (through nonlinear regression). (**e**) Proliferation assay in the fifth human primary ATC cell cultures with *BRAF^V600E^* mutation. The IC50 was 4.22 µM (through nonlinear regression). Bars represent the mean ± SD. ns: not significant (*p* > 0.05) * *p* < 0.05 vs. control as per Bonferroni–Dunn test.

Increasing concentrations of cabozantinib (1, 5, 10, 50 and 100 μM) were tested in human primary ATC cell cultures showing a significant decrease in viability/proliferation of the treated cells with respect to control (*p* < 0.05) (Figure 2a–e). We report the IC50 of all the tested cells in Table 2 (see also Figures S8 and S9), and it was lower in the human primary cells and in the continuous cell line 8305C harboring the *BRAF^V600E^* mutation with respect to the IC50 found in the human primary cells and in the continuous cell line CAL62 harboring the *NRAS* mutation.

### 3.2. Cabozantinib Showed a Significant Proapoptotic Effect in Continuous and Human Primary ATC Cell Cultures

We tested the proapoptotic effect of cabozantinib in primary ATC cells. At increasing concentrations of cabozantinib (1, 5, 10, 50, and 100 μM) corresponds an increase in apoptotic cells.

**Figure 3:**
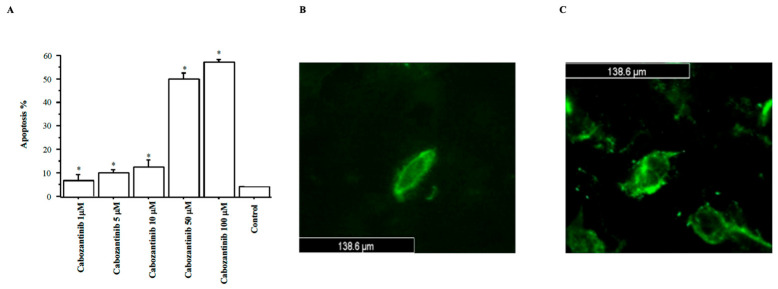
(**A**) Apoptosis in human primary ATC cell cultures after treatment with cabozantinib for 2 days (by Hoechst 33342). Bars represent the mean ± SD, and they were analyzed through one-way ANOVA (with Newman–Keuls multiple comparisons test and a linear trend test) (* *p* < 0.001 vs. control). (**B**) Apoptosis in human primary ATC cell cultures without treatment (through annexin V staining) (final magnification, 400X). (**C**) Apoptosis in human primary ATC cell cultures after treatment with cabozantinib 50μM for 2 days (through annexin V staining) (final magnification, 400X).

The treatment with 1 μM cabozantinib is related to 6.5% of apoptotic cells and the apoptotic rate increases at the higher cabozantinib concentrations reaching the 12.4%, 50%, and 56% apoptotic rate, respectively (*p* < 0.001, ANOVA; Figure 3A). Apoptosis is confirmed through annexin V staining under a treatment with cabozantinib 50 μM; we have also reported the negative control without treatment (Figure 3B,C).

**Figure 4:**
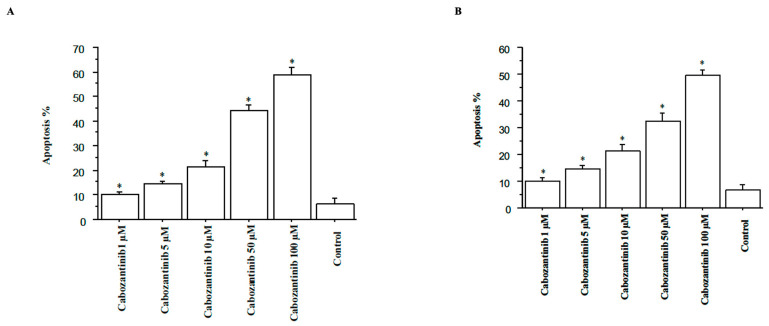
(**A**) Apoptosis in 8305C cells after treatment with cabozantinib for 2 days (through Hoechst 33342). (**B**) Apoptosis in CAL-62 cells after treatment with cabozantinib for 2 days (through Hoechst 33342). Bars represent the mean ± SD, and they were analyzed through one-way ANOVA (with Newman–Keuls multiple comparisons test and a linear trend test) (* *p* < 0.001 vs. control).

Cabozantinib significantly (*p* < 0.05) increased apoptosis in 8305C and CAL62cells (Figure 4A,B).

### 3.3. Low-Dose Cabozantinib Significantly Inhibited Migration in Continuous and Human Primary ATC Cell Cultures

**Figure 5:**
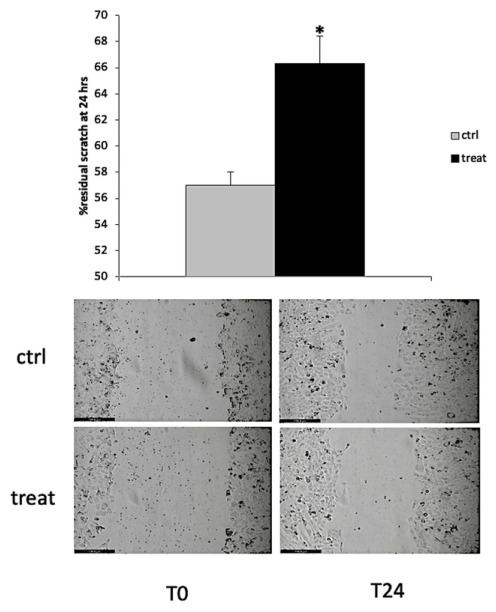
Scratch test in human primary ATC cell cultures after treatment with 1 μM cabozantinib. Data are shown as the mean ± SD. * *p* < 0.05 vs. control as per Student’s *t*-test (final magnification, 20X).

The scratch test showed a significant inhibition of migration in human primary ATC cell cultures with respect to control (*p* < 0.05) at the lowest concentration of cabozantinib (1 μM) (Figure 5).

**Figure 6:**
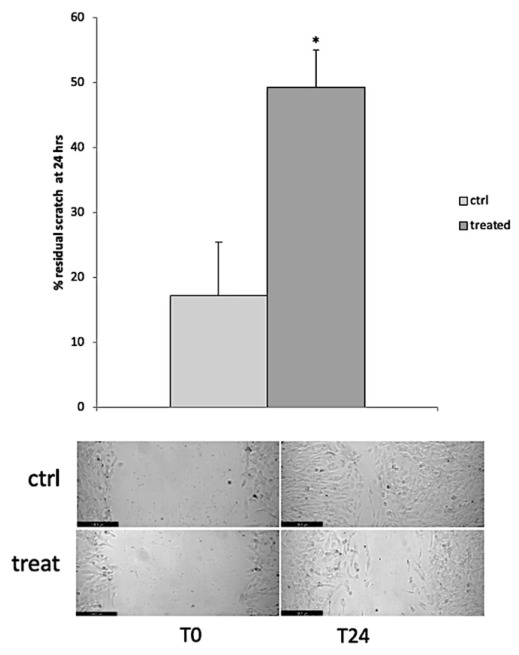
Scratch test in 8305C cells after treatment with 1 μM cabozantinib. Data are shown as the mean ± SD. * *p* < 0.05 vs. control as per Student’s *t*-test (final magnification, 20X).

A reduction in migration has been observed in 8305C (Figure 6), with similar results also obtained in CAL-62.

### 3.4. Cabozantinib Inhibited Akt and ERK1/2 Phosphorylation in Human Primary ATC Cells and Induced the Activation of Bax Protein

**Figure 7:**
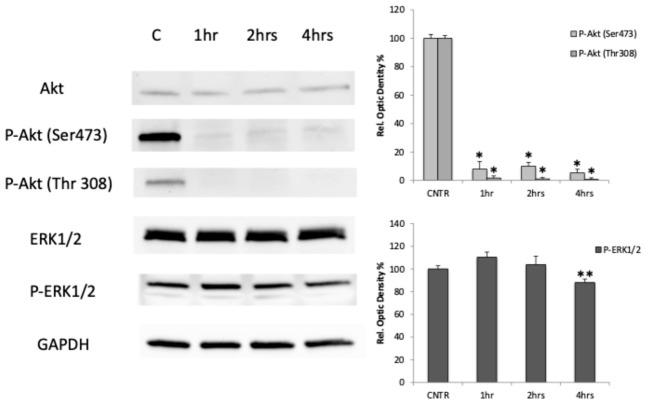
Western blotting analysis in human primary ATC cell cultures after treatment with 50 μM cabozantinib. * *p* < 0.0001; ** *p* < 0.05.

Western blotting analysis showed an inhibition of Akt and ERK1/2 phosphorylation by cabozantinib (50 μM) in human primary ATC cells (Figure 7) (see also Figures S2–S7).

**Figure 8:**
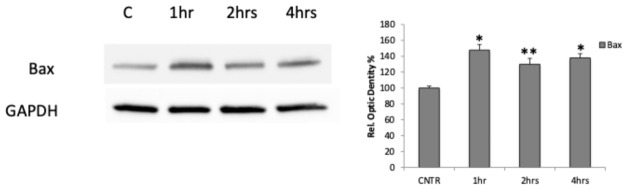
Western blotting analysis in human primary ATC cell cultures after treatment with 50 μM cabozantinib. * *p* < 0.0001; ** *p* < 0.05.

We also confirmed the induction of apoptosis through the activation of Bax protein by cabozantinib (50 μM) in human primary ATC cells (Figure 8) (see also Figure S1).

### 3.5. Cabozantinib Decreased CXCL10 and CCL2 Levels in Human Primary ATC Cell Cultures

In human primary ATC cell cultures, CXCL10 and CCL2 were detectable basally in the supernatants of all samples, as previously shown [34,35].

**Figure 9:**
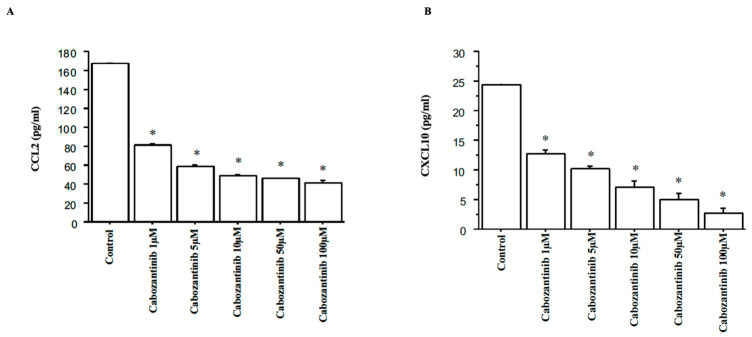
(**A**) CCL2 secretion in human primary ATC cell cultures after treatment with cabozantinib; (**B**) CXCL10 secretion in human primary ATC cell cultures after treatment with cabozantinib. Data are shown as the mean ± SD. * *p* < 0.05 vs. control as per Bonferroni–Dunn test.

The treatment with cabozantinib (1, 5, 10, 50, and 100 μM) led to a decrease in both chemokines with respect to the control (*p* < 0.05) (Figure 9A,B).

## 4. Discussion

The management of patients with ATC remains challenging. Although current therapies can effectively alleviate local symptoms, overall prognosis remains poor, with median survival still exceedingly short [7,46,47]. Advances in the understanding of ATC oncogenesis have expanded the range of therapeutic options targeting the genetic alterations and molecular pathways involved in tumor development and progression [7]. Moreover, a multimodal treatment approach combining surgery, radiotherapy, chemotherapy, molecular targeted drug therapy, and immunotherapy is increasingly being adopted to improve clinical outcomes [48,49,50]. Recently, FDA has approved the use of dabrafenib and trametinib for the treatment of ATC with *BRAF^V600E^* mutation not treatable through surgery or the one that has metastasized [51]. The significant clinical effects observed in vivo [23] have also been confirmed by in vitro studies that have shown an antiproliferative effect of the combined drugs in *BRAF^V600E^* mutated cancer cells of different types and in TC cells [52,53,54]. In vitro and in vivo studies on human primary ATC cells have shown the antineoplastic activity of lenvatinib [18], which was recently approved in Japan for patients with unresectable TC of all histological subtypes [55]. Cabozantinib demonstrated an antineoplastic effect in preclinical and clinical models of different types of cancer [56]. Cabozantinib was approved for metastatic MTC [57], and lately for locally advanced or metastatic DTC, refractory or not eligible to RAI and that has progressed during or after prior systemic therapy [28]. In vitro studies showed the antiproliferative efficacy of cabozantinib in inhibiting cell proliferation in continuous papillary (PTC) cells with RET/PTC1 rearrangement [56]. Its antineoplastic efficacy was also demonstrated by another study on continuous PTC and MTC cell lines [58].

To date, no studies are present in the literature about the in vitro antineoplastic effect of cabozantinib in ATC continuous cell lines, nor in primary ATC cells. Here, we have demonstrated a significant antineoplastic activity of cabozantinib in ATC cell lines (8305C and CAL62); furthermore we have shown its antineoplastic effect in primary ATC cells obtained directly from patients, independently from the presence of specific mutations. Noteworthy the obtained IC50 is lower in the human primary cells and in the continuous cell line 8305C harboring the *BRAF^V600E^* mutation with respect to the IC50 found in the human primary cells and in the continuous cell line CAL62 harboring the *NRAS* mutation. However, we performed our experiments in a small number of samples, and this is in line with other studies on ATC [18,39], since it is an extremely rare disease. Therefore, we are not able to draw definitive conclusions and these findings might be validated in larger, multicenter cohorts or through meta-analysis in future studies. Moreover, in line with other previous studies [34,35] we found high levels of CCL2 and CXCL10 in human primary ATC cell cultures confirming that primary ATC cells are able to produce both Th1 and Th2 chemokines basally, which are involved in the immune response against the tumor. Our results first showed that the treatment with cabozantinib led to a decrease in CXCL10 and CCL2 secretion by ATC cells, suggesting an immunomodulatory effect of the drug. A reduction in chemokine levels in the tumor have a great impact considering that, on the one hand, CXCL10 is active in recruiting cytotoxic T-cells, but on the other, it is a potent recruiter of immunosuppressive populations cells, notably regulatory T-cells (Tregs) and myeloid-derived suppressor cells (MDSCs); in this way it can allow an immune-suppressive environment that can counteract therapeutically recruited effectors [59]. Also CCL2 has a central role in both harmful and protective inflammatory states, including cancer-mediated inflammation. The CCL2/CCR2 axis activation was shown to aggravate the progression of different cancer types by recruiting macrophages with an immune-suppressive phenotype [60].

In conclusion, our results show the efficacy of cabozantinib as antineoplastic and immunomodulatory drug in ATC in vitro, and pave the way for a personalized therapy by using in vitro studies in primary ATC cell in each patient.

## 5. Limitations and Strengths of the Study

Our study aimed at evaluating cabozantinib as an antineoplastic drug for the treatment for ATC in vitro.

A first limitation could be the number of patients enrolled; however, as previously reported, ATC is a rare cancer.

Usually, since cell lines are easy to manage, they are preferred for the in vitro experiments; however they tend to adapt to the in vitro growth conditions losing their thyroid specific characteristics. In addition, genetic analysis showed that several thyroid cell lines are misidentified or cross-contaminated with other cells, failing to closely reproduce the in vivo environment. Differently, although primary human cells have a limited lifespan, allow researchers to investigate not only cells, but also donors’ specific variables, such as gender or medical history, which can be considered during the evaluation of the experimental model [61]. Therefore, we performed in vitro tests with primary human ATC cell cultures that differently to continuous cells are more appropriate in the assessment of the antineoplastic effect of different drugs, because the phenotypic features allow to obtain a better comparison with the original tumor. Then, primary cells can be used for in vitro studies to assess the chemosensitivity of cells of each subject, taking into account that a positive antineoplastic effect obtained in vitro can predict a positive anti-tumoral effect of the drugs in the same patients with a 60% positive predictive value. Furthermore, with in vitro studies the negative predictive value is of 90%, which means that a negative antineoplastic effect on the primary cells can predict an absent anti-tumoral effect in the same patients with this percentage [39,62,63]. A negative predictive value is important in avoiding the administration of ineffective drugs in non-responsive patients, thus avoiding harmful side effects or life-threatening complications.

A further limitation is the in vitro nature of the study that is not able to fully recreate the complex in vivo tumor microenvironment. However, as previously outlined, we sought to overcome this limitation by using human primary ATC cells and in the future we aim to conduct further in vitro studies able to mimic the in vivo tumor by analyzing the interactions with other cells in the tumor niche by using co-cultures and to validate our preclinical results with in vivo study in nude mice.

Overall, these findings are promising and suggest a future in vivo evaluation of cabozantinib in patients with ATC. Indeed, in vitro tests are a good model in predicting the clinical activity of the drug in vivo.

## 6. Conclusions

Anaplastic thyroid cancer is a highly aggressive malignancy, and current therapeutic strategies remain largely ineffective in significantly improving patient outcomes.

We tested the antineoplastic activity of cabozantinib in vitro, and, in the first place, we have shown that it is able to inhibit cell proliferation significantly, increasing apoptosis and inhibiting the migration in continuous and in human primary ATC cells. Moreover, cabozantinib decrease the secretion of the Th1 (CXCL10) and Th2 (CCL2) chemokines by ATC cells, suggesting an immunomodulatory effect of the drug.

In our opinion, these results are very promising for a future in vivo evaluation of cabozantinib in patients with ATC; moreover our study sheds a light on the use of in vitro studies for a future personalization of the therapy that can allow the administration of the appropriate and effective treatments that avoid ineffective and potentially harmful drugs. Indeed, a personalized approach for each patient can be achieved by using primary human ATC cells obtained from each patient, and therefore, with in vitro studies, it is possible to assess the chemosensitivity of the cells of each subject.

Therefore, these results, in our opinion, can open the way to a future personalization of the therapies through in vitro studies of primary ATC cells from each patient.

## Figures and Tables

**Table 1:** Clinical characteristics of the five patients from whom primary cells were derived.

Human Primary Anaplastic Thyroid Cancer Cells	Gender	Age	Histological Diagnosis	Immunophenotypic Investigations	Cell Mutation Status
1°	M	63	Morphological and immunophenotypic findings compatible with undifferentiated (anaplastic) thyroid carcinoma, extensively sclerotic, infiltrating the lax perithyroid tissues; presence of vascular invasion (A1-7). pT3aNx.	Cytokeratins CKpan e CKCAM5.2 focal immunoreactivity PAX8 positive; TTF-1 negative.	NRAS+
2°	F	66	Anaplastic carcinoma, extensively necrotic, infiltrating the lax perithyroid tissues; presence of tumor emboli, multiple lymph node metastases. Multiple microfoci of papillary carcinoma. pT4bN1b.	Cytokeratin CKCAM5.2: positive. Thyroglobulin and TTF-1: district-wise positive.	BRAF^V600E+^
3°	F	76	Morphological and immunophenotypic findings consistent with poorly differentiated carcinoma (4.5 cm) with anaplastic (undifferentiated) features, extensively necrotic, infiltrating the thyroid parenchyma and lax perithyroid tissues; diffuse neoplastic embolization. Right laterocervical lymph node metastases. Esophageal tissue infiltration. pT4aN1bMx.	Cytokeratin CKCAM5.2: positive. Thyroglobulin and TTF-1: district-wise positive.	No mutations among those analyzed.
4°	M	68	Morphological findings compatible with undifferentiated (anaplastic) carcinoma infiltrating muscle tissue (A1-2). pT4aN1b.	Cytokeratin CKCAM5.2: positive. Thyroglobulin and TTF-1: negative.	No mutations among those analyzed.
5°	F	53	Anaplastic carcinoma infiltrating the perithyroid lax tissue and muscle, with widespread bilateral vascular embolization. Lymph node metastases. pT4bN1aMx.	Cytokeratin CKCAM5.2: positive. Thyroglobulin and TTF-1: negative.	BRAF^V600E+^

**Table 2:** IC50 in human primary cells and in the continuous cell lines.

Continuous and Human Primary Cells	IC50
CAL-62	18.64
8305C	3.30
1st human primary cells	36.04
2nd human primary cells	6.16
3rd human primary cells	19.90
4th human primary cells	32.22
5th human primary cells	4.22

## Data Availability

The data presented in this study are available within the article.

## Supplementary Materials

The following supporting information can be downloaded at: https://www.mdpi.com/article/10.3390/cancers18152379/s1, Figure S1: Western blotting analysis of Bax; Figure S2: Western blotting analysis of p-Akt (Ser 473); Figure S3: Western blotting analysis of p-Akt (Thr 308); Figure S4: Western blotting analysis of Akt; Figure S5: Western blotting analysis of ERK1/2; Figure S6: Western blotting analysis of pERK1/2; Figure S7: Western blotting analysis of GAPDH; Figure S8: IC50 graphs of continuous cell line CAL-62 and 8305C (through nonlinear regression); Figure S9: IC50 graphs of the 5 ATC human primary cells (through nonlinear regression).
