# Supplementary material for: Antineoplastic and Immunomodulatory Effects of Cabozantinib in Continuous and Primary Human Anaplastic Thyroid Cancer Cells
Source: Cancers (Basel). 2026 Jul 23;18(15):2379. doi: 10.3390/cancers18152379 (PMC13465927; doi:10.3390/cancers18152379)
Supplement: Supplementary file 1 [file cancers-18-02379-s001.zip › Supplementary Figure S6. Western blotting analysis of pERK12.pdf]

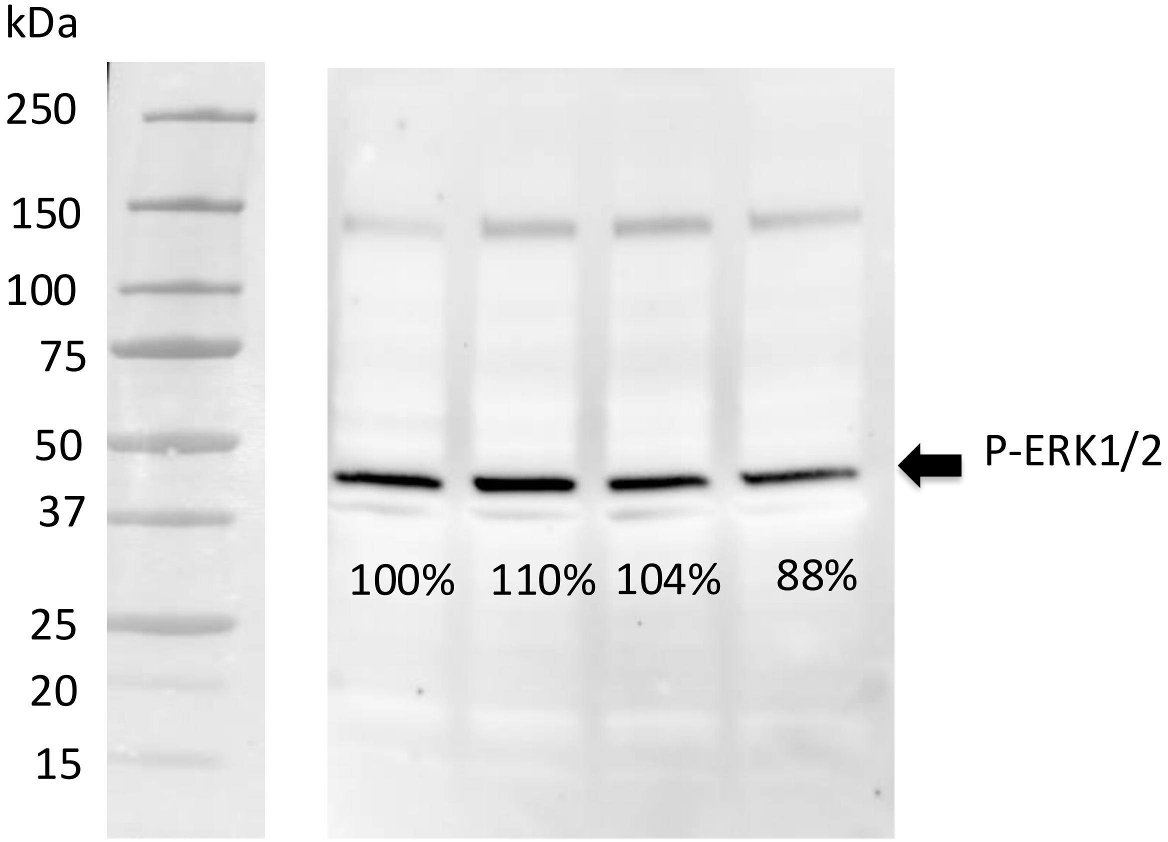
kDa
250
150
100
75
50
37
25
20
15
P-ERK1/2
100%
110%
104%
88%
