# Supplementary material for: Antineoplastic and Immunomodulatory Effects of Cabozantinib in Continuous and Primary Human Anaplastic Thyroid Cancer Cells
Source: Cancers (Basel). 2026 Jul 23;18(15):2379. doi: 10.3390/cancers18152379 (PMC13465927; doi:10.3390/cancers18152379)
Supplement: Supplementary file 1 [file cancers-18-02379-s001.zip › Supplementary Figure S7. Western blotting analysis of GAPDH.pdf]

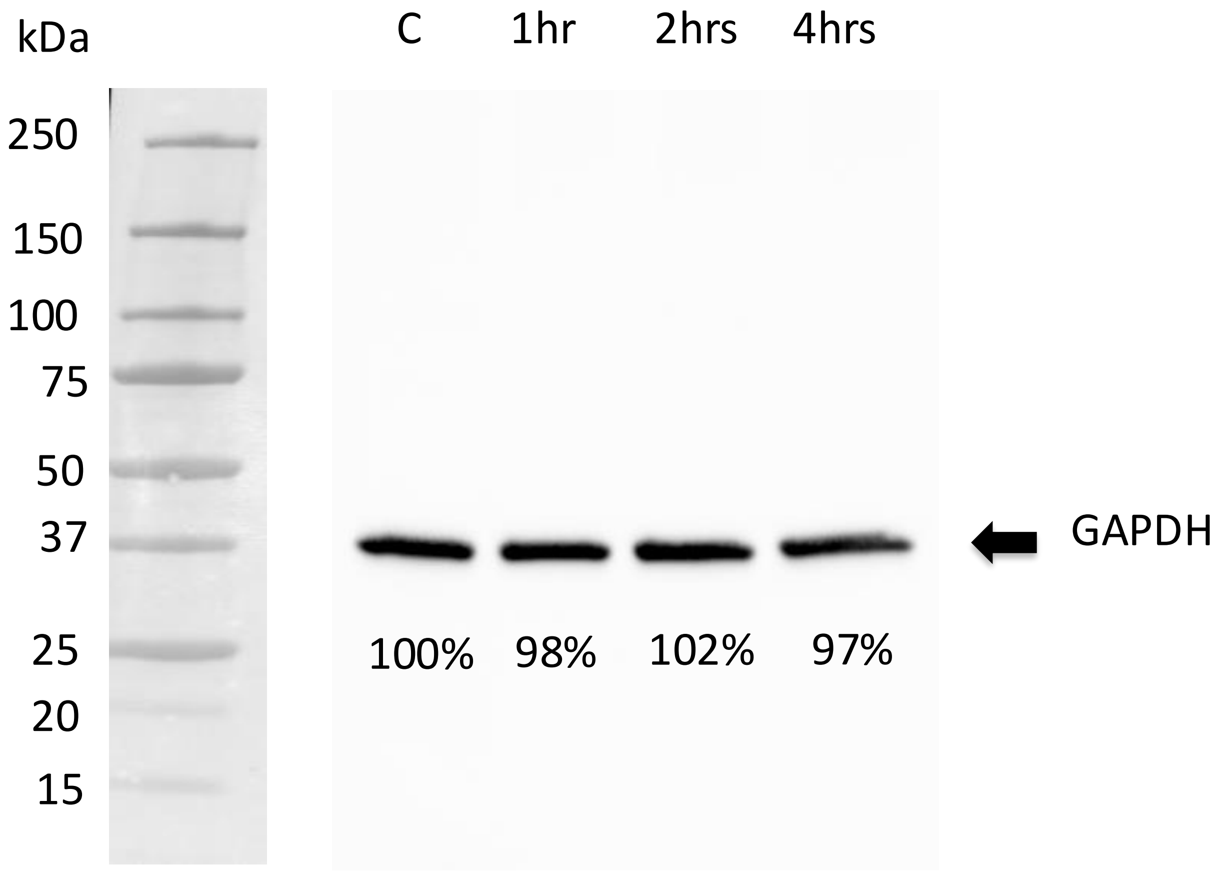
kDa
250
150
100
75
50
37
25
20
15
C
1hr
2hrs
4hrs
GAPDH
100%
98%
102%
97%
