# Supplementary material for: Antineoplastic and Immunomodulatory Effects of Cabozantinib in Continuous and Primary Human Anaplastic Thyroid Cancer Cells
Source: Cancers (Basel). 2026 Jul 23;18(15):2379. doi: 10.3390/cancers18152379 (PMC13465927; doi:10.3390/cancers18152379)
Supplement: Supplementary file 1 [file cancers-18-02379-s001.zip › Supplementary Figure S8. IC50 graphs of continuous cell line CAL-62 and 8305C (through nonlinear regression).pdf]

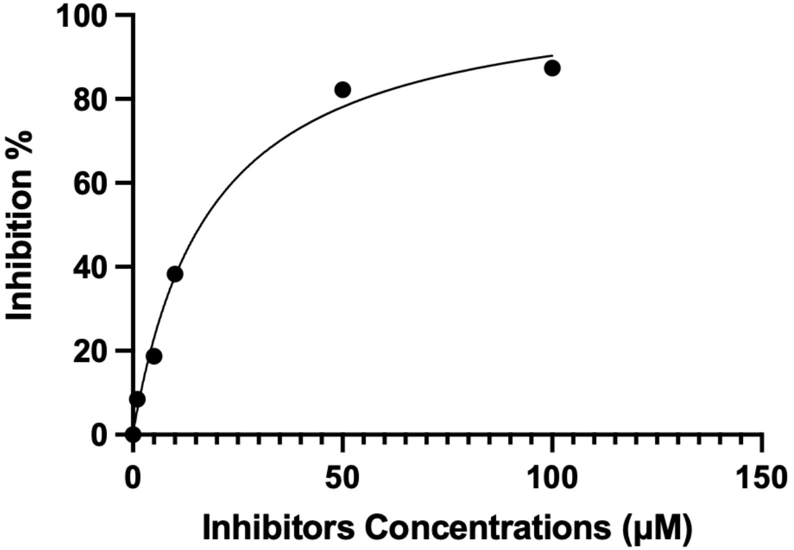


IC50 CAL-62

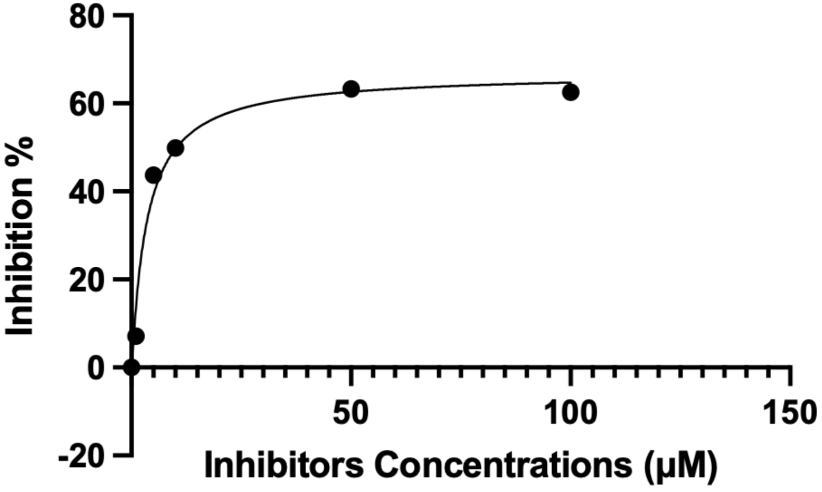


IC50 8305C
