# Supplementary material for: Antineoplastic and Immunomodulatory Effects of Cabozantinib in Continuous and Primary Human Anaplastic Thyroid Cancer Cells
Source: Cancers (Basel). 2026 Jul 23;18(15):2379. doi: 10.3390/cancers18152379 (PMC13465927; doi:10.3390/cancers18152379)
Supplement: Supplementary file 1 [file cancers-18-02379-s001.zip › Supplementary Figure S9. IC50 graphs of the 5 ATC human primary cells (through nonlinear regression).pdf]

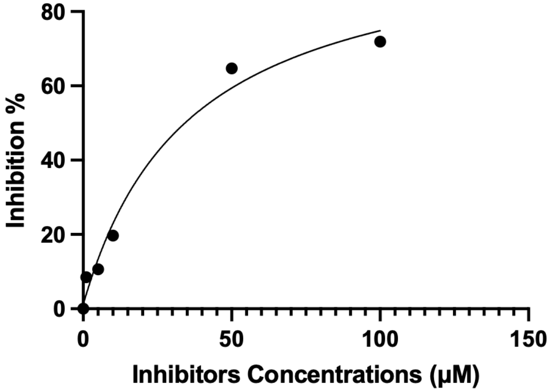

IC50 - 1st human primary cells

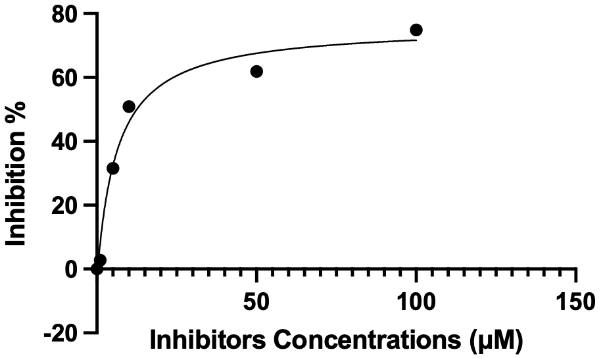

IC50 – 2nd human primary cells

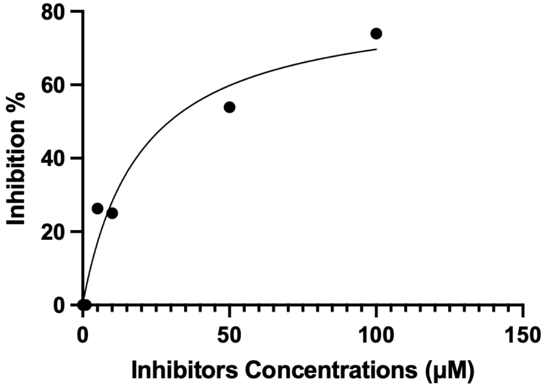

IC50 – 3rd human primary cells

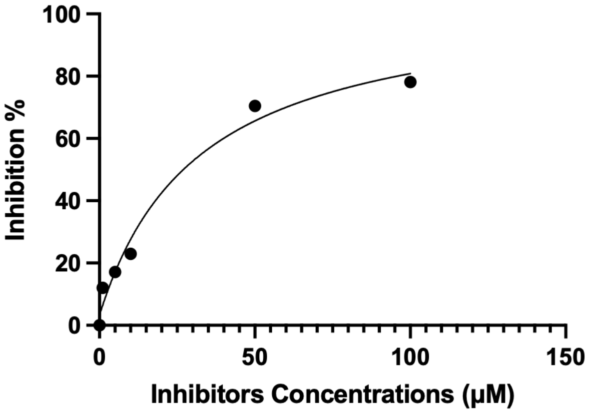

IC50 – 4th human primary cells

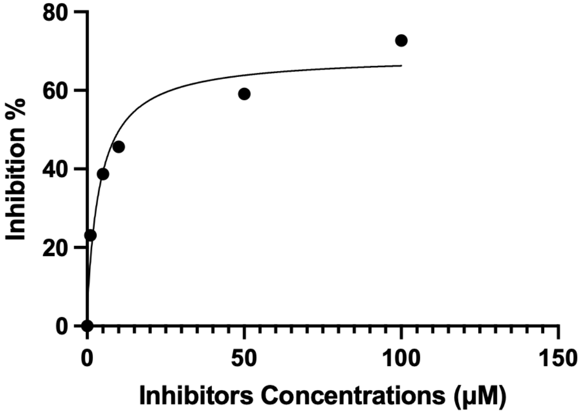

IC50 – 5th human primary cells
